# Self-reported surgeon health behaviours: A multicentre, cross-sectional exploration into the modifiable factors that impact surgical performance with the association of surgeons in training

**DOI:** 10.1016/j.amsu.2021.102299

**Published:** 2021-04-27

**Authors:** Dale F. Whelehan, Tara M. Connelly, Joshua R. Burke, Eva M. Doherty, Paul F. Ridgway

**Affiliations:** a Department of Surgery, School of Medicine, Trinity College Dublin, The University of Dublin, Ireland; b Department of Surgery Tallaght University Hospital, Ireland; c Department of Gastrointestinal Surgery, Leeds Teaching Hospitals, NHS Trust, United Kingdom; d Department of Surgery, Royal College of Surgeons in Ireland, Ireland

**Keywords:** Sleep deprivation, ASiT, Surgical performance, Fatigue, Performance management

## Abstract

**Introduction:**

Surgeons regularly educate patients on health promoting behaviours including diet, sleep and exercise. No study thus far has explored surgeons’ personal compliance with these health behaviours and their relationship with surgical performance. The primary outcomes of this study were self-reported health, health related behaviours, wellbeing, fatigue and surgical performance.

**Methods:**

A survey of validated themes on health related behaviours, workplace variables and performance was distributed to surgical trainees and consultants in the UK and Ireland through the Association for Surgeons in Training (ASiT). Non-parametric analysis was used to determine inferential associations.

**Results:**

Ninety five surgeons (51.5% female, 39.9% registrars) completed the survey. 94% and 74% reported ‘good’ or better overall health and mental wellbeing respectively. The majority (54.7%) reported inconsistent sleep patterns. Less than a quarter engage in regular exercise. Sixty two and 64.2% reported being regularly fatigued and bothered by feelings of anxiety and/or depression respectively. Poor self-reported health and wellbeing were associated with poorer reported off-call performance (p < .01). Higher levels of fatigue negatively impacted self-reported surgical and non-surgical task proficiency (p < .01).

**Discussion and conclusion:**

Surgeons reported high levels of overall health. However, healthy behaviours around sleep, diet and exercise were not consistently reported. Fewer reported good mental health and emotional well-being. Self-reported health behaviours including sleep and physical activity were associated with surgical performance. Strategies to improve modifiable lifestyle factors which will optimise physical health, mental wellbeing and levels of fatigue may optimise surgical performance.

## Highlights

• 94% and 74% respectively reported having at least ‘good’ health and well-being.
• A majority reported being regularly fatigued and inconsistent sleep patterns.
• Poorer health and wellbeing were associated with poorer performance off-call (p < .01).
• Higher levels of fatigue negatively impacted technical and non-technical performance.
• Improving modifiable factors to optimise health and wellbeing may improve performance.

## 1 Introduction

The duties of a surgeon are multifaceted and include educating patients on lifestyle factors known to impact health outcomes such as stress, diet, sleep and exercise. The level of compliance with healthy lifestyle behaviours in surgeons, however, remains unknown and raises the question: ‘Do surgeons practice what they preach?‘. Research has found higher levels of alcohol consumption and lower levels of health enhancing physical activity [1] in Irish cohorts but their impact on performance remains unknown. Efforts exist to optimise surgical performance and to standardise organisations' practice, such as the World Health Organisation surgical checklist [2] and human factors awareness training [3,4]. Work in reducing cultural paternal hierarchies in surgery [5] is also ongoing. However, a dearth of research on the influence of individuals' behaviours and approaches to work in surgery exists [6,7]. No study thus far has exclusively explored surgeons' self-reported health-behaviours and their relationship with surgical performance including error-making.

Fatigue is defined as an overwhelming sense of tiredness, lack of energy and impaired physical and/or cognitive functioning [8]. It is one of the greatest risks to performance decrement [9]. Fatigue is linked to slower reaction times [10], the inability to recognise error [11] and a reduction in adherence to procedural place-keeping [12]. Emotional affect is also altered in fatigued states [13]. This may impact on non-technical skill performance such as patient empathy, shared decision-making and appropriate patient management. It may also impact on collegiality amongst peers and teamworking [14]. The Institute of Medicine's report *To err is human* found that surgical errors were the second highest cause of error-related deaths within healthcare [15]. Many of these surgical errors (22%) may be preventable [16]. and could be associated with individuals' performance. Reducing fatigue levels within surgeons may act as the mediator between health, wellbeing and performance outcomes. Recognising that levels of fatigue are transient, identifying the significant lifestyle and work-behaviours which influence fatigue in surgery may optimise surgical performance.

The aim of this study is to explore relationships levels of self-reported health behaviours and work factors, with overall health, mental wellbeing, level of fatigue, and self-reported surgical performance. The specific objectives of this research were to:

● To explore surgeons' adherence to gold standard guidelines on healthy lifestyle factors
● To explore surgeons self-reporting of work-related factors
● To investigate trends between overall health, mental wellbeing and levels of fatigue and surgical performance

## 2 Methods

This study has been reported in line with the STROCSS criteria [17]. The study was registered with ClinicalTrials.gov (Identifier: NCT04677036).

### 2.1 Survey design

**Fig. 1:**
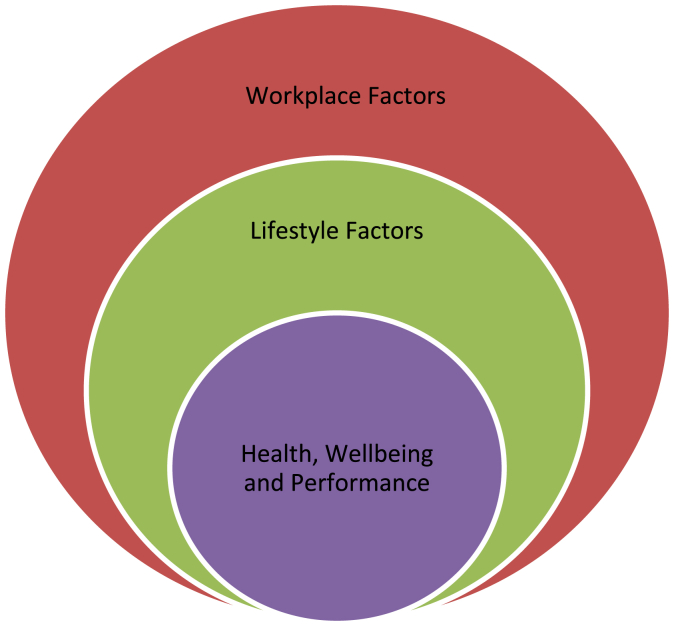
Relationship between the constructs.

A validated survey (*Appendix A*) was developed by the research team in accordance with the Burns criteria on design and conduct of self-administered clinician surveys [18] attached in *Appendix B* based on the constructs seen in Fig. 1, Table 1 and Table 2.

**Table 1:**
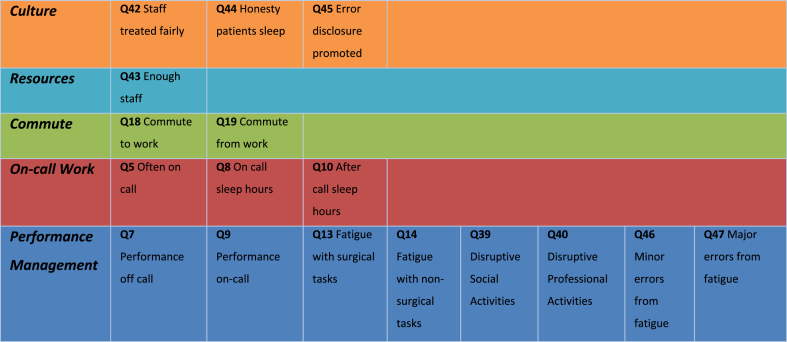
Work Factors with Performance outcomes and associated questions.

**Table 2:**
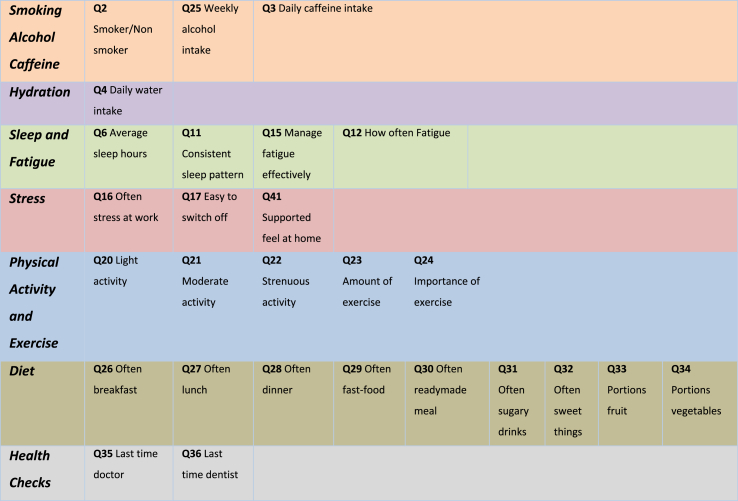
Lifestyle Factors and associated questions.

### 2.2 Research participants

As a cohort study, surgeons from the Association for Surgeons in Training (ASiT) were invited to complete the survey by email invitation and through social media dissemination for purposive sampling. Study approval was obtained from the Joint Research Ethics Committee (JREC) of Tallaght University Hospital and St. James Hospital (2019-07). The survey was open between June–August 2020. All participants provided informed consent and anonymously completed the survey. No financial reward was given. Based on previous published research on surgeons conducted by some of the research team [19], a response rate of between 80 and 100 responses is reflective of average survey response in this domain of research. A reminder email was distributed after 4 weeks.

### 2.3 Statistical analysis

Data was analysed for normal distribution and statistical tests were applied using Statistical Package for Social Sciences (SPSS, version 25). Non-parametric tests were used as the data violated the assumptions of normality. Mann-Whitney U and Kruskal Wallis testing was used to determine significant differences between demographic variables. A matched control of Irish physiotherapists was used to draw comparisons between professions. Validity and reliability tests i.e. Spearman rho and Spearman-Brown Cronbach alpha were also applied to measure associations between variables.

## 3 Results

### 3.1 Demographics

Ninety five surgeons responded, including the pilot cohort. Demographics were collected for 66 (69.5% of respondents) of participants (Table 3). The discrepancy is due to the inclusion of results from the 29 surgeons in the pilot group for whom extensive demographics were not collected (see Table 4).

**Table 3:** Demographic breakdown of participants.

Demographic	Number (Percentage)
Gender	
Male	32 (48.5)
Female	34 (51.5)
Age	
18–24	1 (1.5)
25–34	31 (47)
35–44	26 (39.4)
45–54	8 (12.1)
Length of Time Since Graduation (years)	
≤5	16 (24.2)
6–10	19 (28.8)
11–16	17 (25.8)
17–22	9 (13.6)
≥23	5 (7.6)
Job Title	
Intern/FY1-2	8 (8.4)
SHO/CT1-2	15 (15.8)
Registrar/ST1-3+	37 (38.9)
Consultant	28 (29.5)
Other	7 (7.4)
Sector of Employment	
Public	90 (94.7)
Private	5 (5.3)
Specialty	
General	65 (68.4)
Oral and Maxillofacial	5 (5.3)
Otolaryngology	1 (1.1)
Plastic	4 (4.2)
Trauma and Orthopaedics	11 (11.6)
Urology	5 (5.3)
Vascular	3 (3.2)
Gynaecology	1 (1.1)
Region of Work	
England	23 (24.2)
Wales	3 (3.2)
Scotland	2 (2.1)
Northern Ireland	1 (1.1)
Ireland	62 (65.3)
Other	4 (4.2)

**Table 4:**
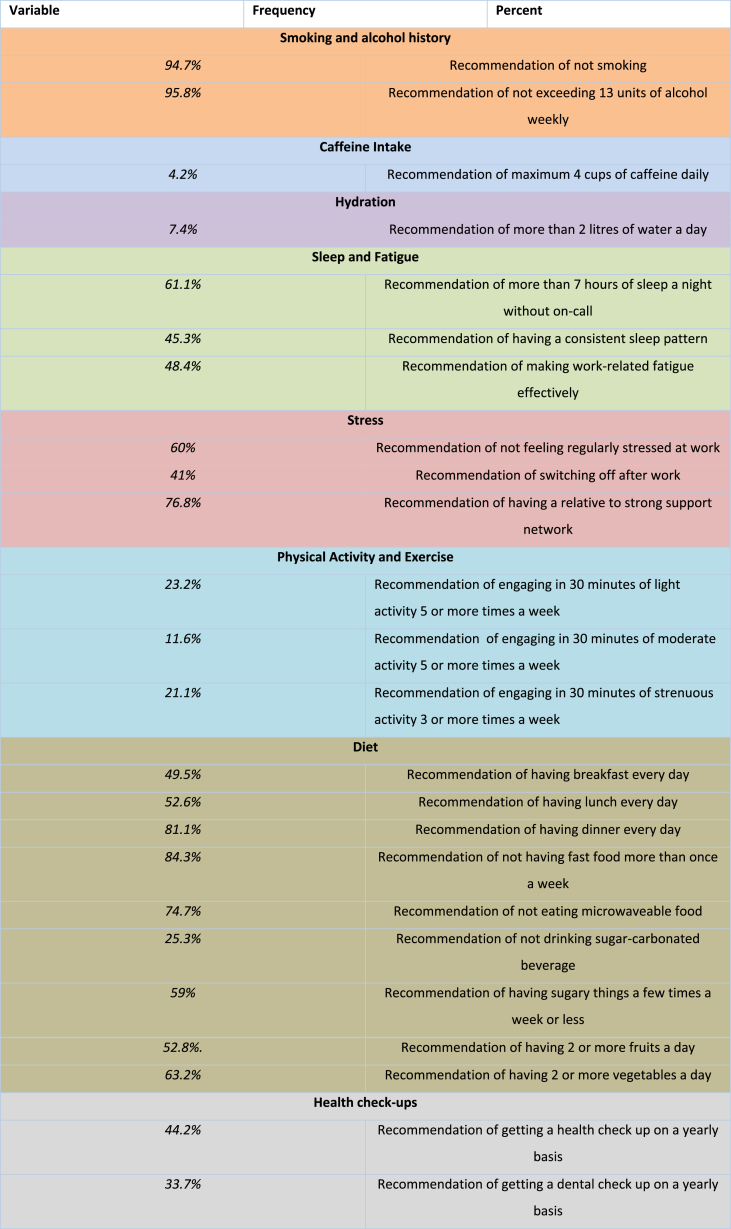
Lifestyle Factors Compliancy in Surgery.

**Table 5:**
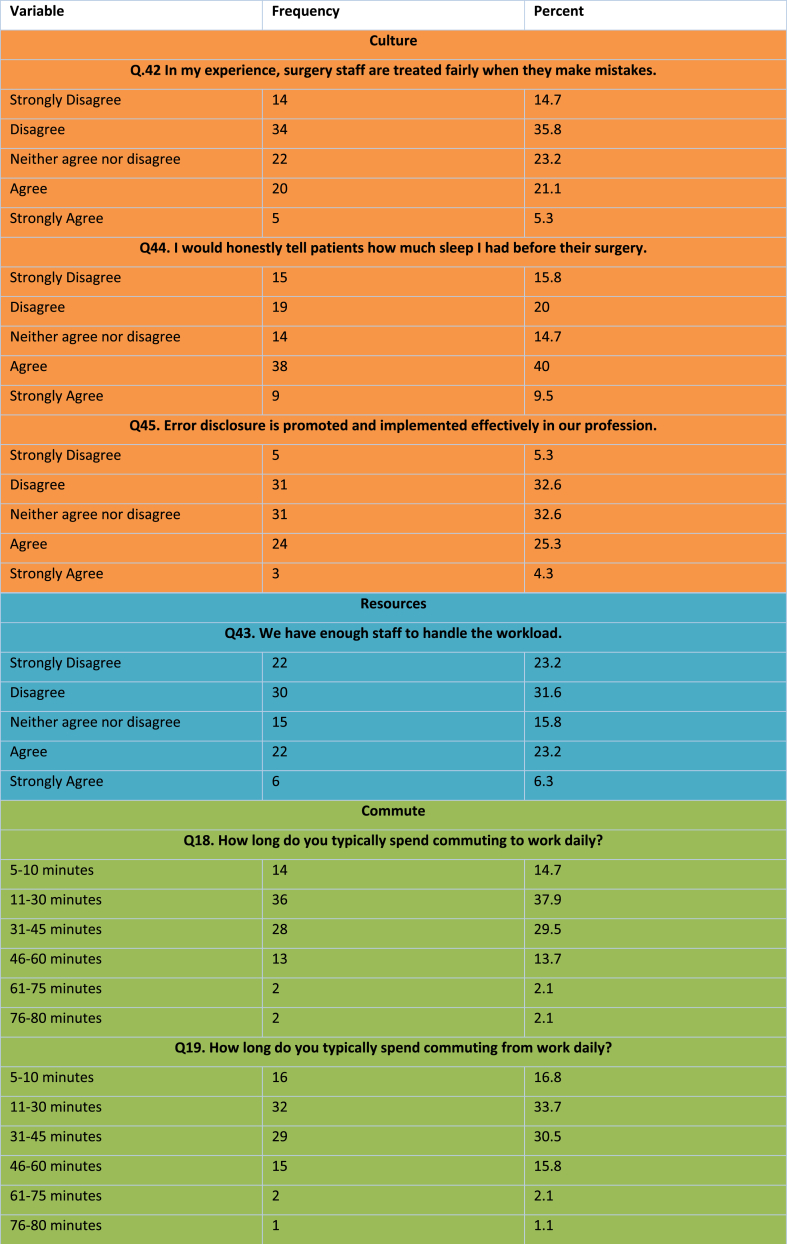

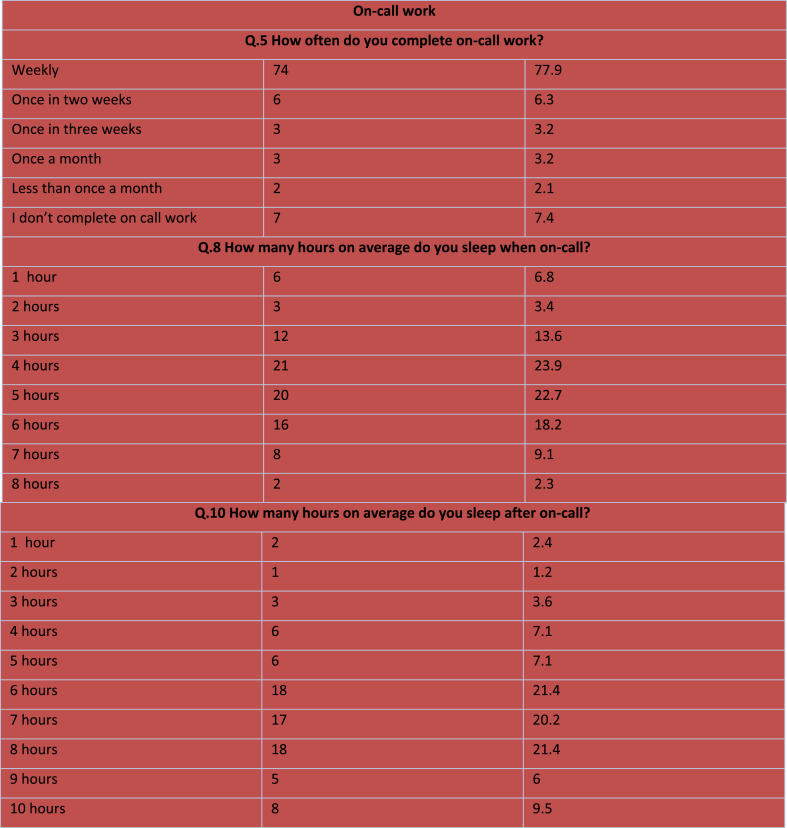
Work Factors in Surgery.

There was variation regarding regularity of on-call work depending on the specialty with general surgery and vascular reporting the greatest amount of weekly on-call work, while oral and maxillofacial surgery reported the least (median of once a week vs once every three weeks, p = .019). Female surgeons were more likely to report feeling regularly stressed work (94% vs 43.8% in males, p = .010). Professional title influenced levels of sleep on-call with interns sleeping the least (median of 3.5 h) while consultants slept the most (median of 6 h).

### 3.2 Surgeons’ health and wellbeing

**Fig. 2:**
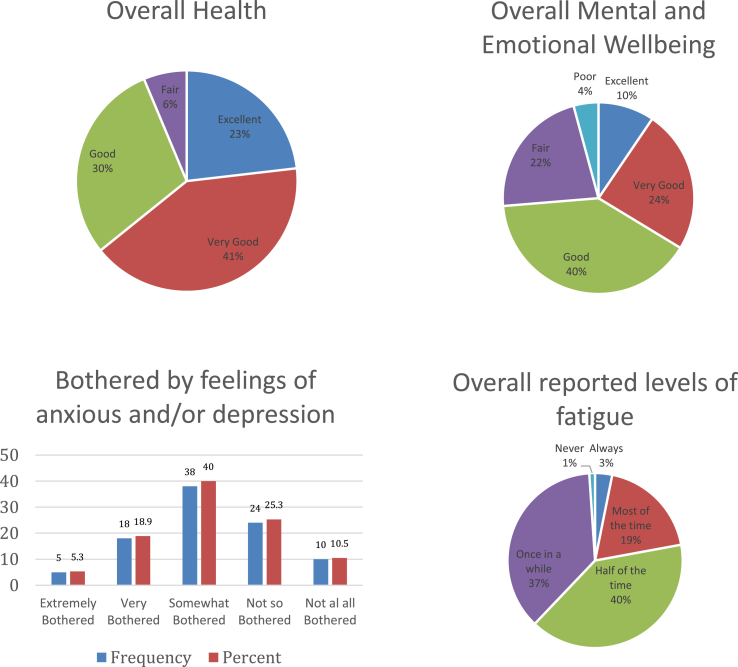
Self-reported levels of overall health, wellbeing and fatigue in surgeons.

The majority (94%) reported overall health that was at least ‘good’ Fig. 2. No respondents reported poor overall health. Seventy four percent reported overall mental and emotional well-being that was at least good (‘good’ = 40%, ‘very good’ = 24%, ‘excellent’ = 10). Forty percent reported being ‘somewhat bothered’ by anxious feeling and/or depression in the last month. Sixty-two percent reported feeling fatigued half of the time or more.

When compared with a cohort of physiotherapists, surgeons report poorer overall health (p < .001), poor mental and emotional wellbeing (p < .001) and being more often fatigued (p < .001) (see Table 5, Table 6).

A breakdown in the distribution of scores is attached in Appendix F. When compared with a cohort of physiotherapists, surgeons report sleeping less overall (p < .001), having more inconsistent sleep (p < .001), not managing fatigue as effectively (p < .031), engaging in less exercise and physical activity (p = .002), having more irregular eating patterns (p < .001) and not receiving health check-ups as regularly (p < .001).

### 3.3 Work related factors

When compared with a cohort of physiotherapists, surgeons report not feeling treated as fairly (p < .001), being less honest to patients (p < .001), not feeling error disclosure is promoted in the workplace (p = .006) and being on-call more often (p < .001).

### 3.4 Performance outcomes

**Table 6:** Performance outcomes in Surgery.

Performance Management		
Q39. How disruptive were your physical health or emotional problems to your normal social activities with family, friends, neighbours, or groups?		
Extremely Disruptive	2	2.1
Very Disruptive	7	7.4
Somewhat Disruptive	29	30.5
Not so Disruptive	43	45.3
Not at all Disruptive	14	14.7
Q.40 How disruptive were your physical health or emotional problems to your normal professional activities?		
Extremely Disruptive	1	1.1
Very Disruptive	7	7.4
Somewhat Disruptive	21	22.1
Not so Disruptive	42	44.2
Not at all Disruptive	24	25.3
Q.7 How would you rate your overall daily work performance when you're not on-call?		
Excellent	15	15.8
Very Good	55	57.9
Good	21	22.1
Fair	4	4.2
Q.8 How would you rate your overall daily work performance when on-call?		
Excellent	7	7.9
Very Good	30	33.7
Good	38	42.7
Fair	13	14.6
Poor	1	1.1
Q.13 In a typical week, how often do you feel fatigue negatively impacts your ability to perform surgical tasks optimally?		
Most of the time	4	4.2
Half of the time	15	15.8
Once in a while	63	66.3
Never	13	13.7
Q14. In a typical week, how often do you feel fatigue negatively impacts your ability to perform non-surgical professional tasks optimally?		
Most of the time	10	10.5
Half of the time	20	21.1
Once in a while	59	62.1
Never	6	6.3
Q46. I have made minor work-errors as a result of fatigue.		
Strongly Disagree	4	4.2
Disagree	17	17.9
Neither Disagree or Agree	10	10.5
Agree	59	62.1
Strongly Agree	5	5.3
Q47. I have made major work-errors as a result of fatigue.		
Strongly Disagree	21	22.1
Disagree	50	52.6
Neither Disagree or Agree	14	14.7
Agree	7	7.4
Strongly Agree	3	3.3

When compared with a cohort of physiotherapists, surgeons report poorer overall performance (p = .006), greater disruption to social and professional activities due to emotions (p = .001), and making more errors due to fatigue (p < .001).

### 3.5 Variable trends associated with overall health, mental/emotional wellbeing and fatigue levels

A linear trend exists between decreased fatigue and increased overall health and wellbeing. (*See Appendix F*). The lifestyle factors of having ‘consistent sleep’ and ‘feeling supported’ were common across better reported overall health, wellbeing and lower reported fatigue.

Better reported overall health, wellbeing and lower reported fatigue had common trends with decreased disruption to professional activities, better on-call performance and decreased impact of fatigue negatively impacting non-surgical professional tasks.

## 4 Discussion

This survey found ninety-four percent of surgeons reported overall health at ‘good’ or above. Discrepancies exist between overall health reporting with emotional and mental well-being reported (74% ‘good’ or above), reporting of anxious and depression (64.2%) and feeling fatigued at least half the time (62%). When compared with a cohort of physiotherapists, surgeons reported significantly worse health and wellbeing outcomes which translated across to lifestyle and work factors, as well as having negative implications for performance outcomes which are elaborated discussed below.

### 4.1 Lifestyle

In this unique, anonymous survey based setting, surgeons demonstrated variable levels of self-reported compliance with healthy behaviours. Overall, surgeons reported high levels of compliance with non-smoking, acceptable alcohol consumption and number of hours of sleep during non-call nights. However, adherence to recommendations on daily caffeine intake [20] were very low (4.2%). Regular meal consumption, particularly breakfast and lunch, was only seen in half of respondents. While most did not compromise their dietary intake with regular processed food or fast food, recommended vegetable and fruit intake was low [21]. Working in precarious work conditions which require high levels of alertness may explain why many surgeons sugar intake was higher than recommended.

The high percentage of non-smokers and surgeons within guidelines for weekly alcohol intake [22] found in our study is inconsistent with parallel studies. Previous self-reported studies have found surgeons to have significant alcohol use [23]. It is evident that exercise is an important lifestyle factor for surgeons with over 90% of respondents indicating so. Nonetheless, 84.1% reported they were not getting enough exercise. Less than a quarter of respondents met recommended physical activity guidelines in moderate and strenuous activity for health enhancing benefits [24]. Finally, it would appear that a significant portion of surgeons were not regularly checking in on their own health. This may be the result of time constraints which may reduce opportunities for surgeons to be self-aware of health and wellbeing and mitigate appropriate areas for improvement.

The discussion around the importance of sleep has been at the forefront of health behaviours with increasing links associating sleep deprivation with chronic diseases such as diabetes [25]. While nearly two thirds of surgeons are meeting the National Sleep Foundation guidelines [26] of 7–8 h of sleep, less than half (45.3%) report having a consistent sleep pattern. This argues the mismatch between what is an acceptable sleep quantity, but an unacceptable sleep quality culminating and contributing to nearly half (51.6%) reporting they do not manage fatigue effectively. Sleep deprivation in healthcare practice has been found to be higher than average, and results in an increased cognitive workload [27]. Sleep consistency may also be influenced by personal habits causes such as high caffeine intake but also environmental issues such as the regularity of on-call work which differs from that of the physiotherapy control group. Sleep and effective rest strategies may also be related to intrinsic mindsets towards stress and challenge. In this study, 40% of surgeons reported regularly stressed at work with 59% reported finding it difficult to switch off after work indicating a potential downfall in establishing personal and work boundaries in this occupation.

### 4.2 Work factors

Fatigue from work may act as the mediator between health, wellbeing and performance outcomes. Between 30 and 40% of surgeons report disruptions to their social and professional activities due to physical or emotional health problems on a regular basis. While nearly all surgeons report performing well off-call, this decreases when reporting on-call performance, and doesn't match with the levels of self-reported impact of fatigue impacting on both surgical and non-surgical tasks and error-making. Performance regulation is reduced in fatigued states and in this case may lead surgeons to overestimate their ability and continue to work. This leads to a cycle of activating compensatory psychophysiological mechanisms, which results in increased strain and delayed onset cumulative fatigue [28]. Many studies have found that humans do not adapt to chronic levels of sleep restriction, but rather perform sub-optimally [29] at the detriment to physical and mental health. It has also operational implications for ‘fit for duty’ status of personnel [30].

Environmental stressors such as poor resource availability is highly reported in this surgical cohort with over 70% reporting that they are working with insufficient resources to fulfil their professional duties. Overworked and fatigued staff lead to high absenteeism rates [31] thus placing a greater strain on the healthcare system. Regulatory changes such as the European Working Time Directive have brought seismic changes in work-hours in some disciplines such as medicine. Studies have demonstrated that physicians-in-training were working between 80 and 120 h pre-mandate [32]. While such operational measures have been important to create opportunity for greater fatigue management, the first meaningful step to addressing the issue of sleep deprivation in personnel is increasing self-awareness. Behavioural change is routed in an understanding that autonomy of understanding rationale for changes is required in order to accept that change as a personal responsibility [30]. Most healthcare professionals, despite working in an industry which subjects them to 24-h work, receive no education in sleep science or the importance of sleep in maintaining personal health and occupational performance.

Over three-quarters of surgeons reported completing on-call work on a weekly basis, with only 11.4% meeting the recommended sleep guidelines [26] when doing so. Addressing workplace stressors such as call-rotas to promote surgeons ability to self-regulate work-life balance is also therefore important.

### 4.3 Determining variables trending with health, wellbeing, fatigue and performance

While overall health was highly reported by surgeons, a much lower percentage (10%) of surgeons reported having excellent mental and emotional wellbeing. A sizeable majority (64.2%) reported being bothered by feelings of anxiousness and/or depression at the level of ‘somewhat’ or more in the last month. Lower overall health and higher levels of fatigue were associated with poorer mental health outcomes and performance outcomes.

When exploring the influencing factors associated with a surgeons overall health, mental wellbeing and level of fatigue, there was a combination of lifestyle medicine approaches and work-based influencers which played a significant role. Consistent sleep patterns, feeling supported, engaging in the right amount of physical activity, and consistent eating patterns (including increasing vegetable intake while reducing fast food and ready-made meal consumption) all correlated with positive overall health. Higher levels of fatigue were reported by those who felt they were not managing fatigue effectively as well as those with greater levels of stress at work and those who found it more difficult to establish work-life boundaries. While there were less work-place factors associated with the aforementioned outcomes, supportive cultures were associated with better overall health and lower fatigue.

Performance outcomes strongly demonstrated the impact of positive overall health. Those with better overall health reported a lower level of disruption in their professional activities, better performance both off and on-call, and fatigue less likely to impact on non-surgical tasks. The opposite is true for poorer self-reported health such as non-disclosure of levels of fatigue to patients was greater in those who reported poorer overall health. This has additional implications for patient safety.

### 4.4 Recommendations

Based on the above findings, the authors provide a series of evidence-based recommendations for enhancing surgical performance based off current practices.

1 Caffeine consumption should be strategically taken in times of low alertness to maximise effectiveness but should be limited leading up to times of planned rest [33].
2 Education on sleep hygiene techniques, or provision of psychological therapies such as acceptance and commitment therapy (ACT) or cognitive behavioural therapy (CBT) to tackle insomnia in healthcare workers is likely to be effective in tackling issues of rumination and insomnia.
3 Strategic napping should be encouraged in instances of unavoidable sleep disturbance. These naps should have optimal duration of 15–20 min to reduce sleep inertia and may temporarily alleviate the effects of sleep loss [34]. The provision of appropriate rest facilities is likely to have positive effects in this regard.
4 A robust fatigue management programme within institutions should be implemented, incorporating education and training on fatigue scientific principles. Rostering, underpinned by biomathematical approaches [35] should be used for design of work-rotas and workload models. Management should model work-loads to ensure that surgeons have appropriate workload demands, with variety to ensure mental stimulation, reduce overcapacity and optimise performance. For those returning to work, inclusion of gradual return to graded responsibilities in the workforce, greater flexibility in working hours and annual leave, and provision of childcare facilities on site may assist in promoting better work-life balances. For those near retirement, management should be aware of ageing practitioners, and the potential value in which they play in running efficient healthcare services - while being cognisant and effortful to be non-discriminatory. Practitioners performance, as has been discussed in parallel industries such as surgery, should be assessed on ability and not chronological age [36].
5 Improving surgeons access to coaching services [37] on lifestyle modification and personal development strategies may influence this cohorts behaviours to effectively manage lifestyle-related factorss with the view to optimising surgical performance

### 4.5 Strengths and limitations

As this was an opt-in survey, there was some heterogeneity yet consistency in the findings. The use of non-parametric testing allows for appropriate identification of the key findings. A larger sample size is planned through a redistribution of the survey to draw comparisons between the confounding role of COVID-19, and to allow stronger statistical verification of the findings. The comparisons with physiotherapy show the potential for cross-professional collaboration in optimising performance, while also exploring context specific issues such as culture and institutional approaches to staff well-being. Finally, these findings support the planned intervention of a targeted coaching intervention within departments to individuals to identify areas of their own personal lives and professional lives which can be best optimised to improve overall health, well-being and levels of fatigue. This is an innovative way of tackling on-going issues of resilience, burnout and fatigue in surgeons.

## 5 Conclusion

This study demonstrates associations between modifiable self-reported health behaviours and surgical performance. Addressing individual surgeons’ lifestyle management and occupational stressors which affect personal health, wellbeing and fatigue may lead to optimising surgical performance.

## Source of funding

None

## Ethics

Ethical Approval was given by the Joint Research Ethics Committee, SJH AMNCH.

## Funding

This research did not receive any specific grant from funding agencies in the public, commercial, o not-for-profit sectors.

## Provenance and peer review

Not commissioned, externally peer-reviewed.

## Ethical Approval

Study approval was obtained from the Joint Research Ethics Committee (JREC) of Tallaght University Hospital and St. James Hospital (2019-07).

## Author contribution

DFW – study design, data collection, data analysis, writing.

TMC – study design, data collection, writing.

JB – data collection, writing.

EMD. – study design, data analysis.

PFR. – study design, data analysis.

## Research registration Unique Identifying number (UIN)

1. Name of the registry: ClinicalTrials.gov
2. Unique Identifying number or registration ID: NCT04677036
3. Hyperlink to your specific registration (must be publicly accessible and will be checked): https://clinicaltrials.gov/show/NCT04677036
4. See attached receipt attached as PDF

## Guarantor

Dale F Whelehan.

## Data statement

Summative data statement provided in the text only available for access to protect confidentiality of participants.

## Declaration of competing interest

None.
