# Supplementary material for: Self-reported surgeon health behaviours: A multicentre, cross-sectional exploration into the modifiable factors that impact surgical performance with the association of surgeons in training
Source: Ann Med Surg (Lond). 2021 Apr 27;65:102299. doi: 10.1016/j.amsu.2021.102299 (PMC8111267; doi:10.1016/j.amsu.2021.102299)
Supplement: Multimedia component 1 [file mmc1.pdf]

This is a quick survey to better understand your professional and lifestyle behaviours as well as perceptions of the workplace. The summative results of this survey, in conjunction with other assessments will inform future research initiatives to enhance surgery professional well-being and professional performance. Please answer openly and honestly. The survey should take approximately 5-10 minutes to complete. All answers are anonymous and your IP address is not collected. Completion of this survey is indicative of informed consent and data will be collected if you exit the survey before completion. If you have any queries please don't hesitate to get in touch with the lead researcher.

Thank you for participating.

Lead Researcher
Dale Whelehan,
Discipline of Surgery,
School of Medicine,
Trinity College Dublin
whelehd@tcd.ie.

* I confirm I am a surgical trainee/surgeon.

- ☐ Yes

* What is your current gender identity?

- ○ Male
- ○ Female
- ○ Non-Binary/Third Gender
- ○ Prefer not to say

* What is your age?

- ○ 18-24 years old
- ○ 25-34 years old
- ○ 35-44 years old
- ○ 45-54 years old
- ○ 55-64 years old
- ○ ≥ 65 years old

* How long is it since you first qualified from medicine?

- ○ ≤ 5 years
- ○ 6-10 years
- ○ 11-16 years
- ○ 17-22 years
- ○ ≥ 23 years

* Which of the following best describes your professional title?

- Medical Student
- Foundation Trainee (F1 or F2)
- Trust Doctor/Clinical Fellow/Associate Specialist
- Core Trainee (CT1/CT2 or ST1/ST2)
- Specialist Trainee (ST3+)
- Consultant
- Research
- Management
- Other (please specify)

* What sector of work do you primarily work as a surgeon in?

- Public
- Private
- Other (please specify)

* What surgical specialty best describes the area of work you are in?

- Cardiothoracic
- General
- Neurosurgery
- Oral and Maxillofacial
- Neurology
- Otolaryngology
- Paediatrics
- Plastic
- Trauma and Orthopaedics
- Urology
- Vascular
- Other (please specify)

* What region is your work currently based in?

- England
- Wales
- Scotland
- Northern Ireland
- Republic of Ireland
- Other (please specify)

* In general, how would you rate your overall health?

- Excellent
- Very good
- Good
- Fair
- Poor

* Do you currently smoke cigarettes?

- Yes, I do
- No, I do not

* How many caffeinated drinks do you have each day?

- 0
- 1-2
- 3-4
- 5-6
- 7-8
- ≥ 9

* How many litres of water do you have each day?

- < 500ml
- Between 500ml-1000ml
- Between 1000ml-1500ml
- Between 1500ml-2000ml
- > 2000ml

* How often do you complete on-call work?

- Weekly
- Once in two weeks
- Once in three weeks
- Once in a month
- Less than once in a month
- I don't complete on-call work

* How many hours do you sleep on average each night on a week **without on-call**?

1 12

* In general, how would you rate your overall daily work performance when **you're not on-call**?

- Excellent
- Very good
- Good
- Fair
- Poor

How many hours on average do you sleep **when on-call**? [*Skip if you do not complete on-call work*]

1 12

In general, how would you rate your overall daily work performance when **on-call**? [*Skip if you do not complete on-call work*]

- Excellent
- Very good
- Good
- Fair
- Poor

How many hours on average do you sleep **after on-call**? [*Skip if you do not complete on-call work*]

1 12

* Would you consider your sleep pattern consistent?

- [ ] Yes
- [ ] No
- [ ] Other (please state)

* In a typical week, how often do you feel fatigued at work?

- Always
- Most of the time
- Half of the time
- Once in a while
- Never

* In a typical week, how often do you feel fatigue negatively impacts your ability to **perform surgical tasks** optimally?

- Always
- Most of the time
- Half of the time
- Once in a while
- Never

* In a typical week, how often do you feel fatigue negatively impacts your ability to **perform non-surgical professional tasks** optimally?

- Always
- Most of the time
- Half of the time
- Once in a while
- Never

* I manage work-related fatigue effectively.

- Strongly Disagree
- Disagree
- Neither Agree nor Disagree
- Agree
- Strongly Agree

* In a typical week, how often do you feel stressed at work?

- Always
- Most of the time
- Half of the time
- Once in a while
- Never

* I find it easy to switch off after work.

- Strongly Disagree
- Disagree
- Neither
- Agree
- Strongly Agree

* How long do you typically spend commuting **to work** daily?

- 5-10 minutes
- 11-30 minutes
- 31-45 minutes
- 46-60 minutes
- 61-75 minutes
- 76-80 minutes
- Greater than the above

* How long do you typically spend commuting **from work** daily?

- 5-10 minutes
- 11-30 minutes
- 31-45 minutes
- 46-60 minutes
- 61-75 minutes
- 76-80 minutes
- Greater than the above

* How many times in the average week do you engage in 30 minutes of **light activity** (i.e. leisurely walking, gardening, cleaning around the house)?

- 0
- 1
- 2
- 3
- 4
- 5 or more

* How many times in the average week do you engage in 30 minutes of **moderate activity** (i.e. brisk walking, light bicycling)?

- 0
- 1
- 2
- 3
- 4
- 5 or more

* How many times in the average week do you engage in 30 minutes of **strenuous activity** (i.e. running or jogging)?

- 0
- 1
- 2
- 3
- 4
- 5 or more

* Do you feel you get too much exercise, too little exercise, or about the right amount of exercise?

- Much too much
- Somewhat too much
- Slightly too much
- About the right amount
- Slightly too little
- Somewhat too little
- Much too little

* How important is exercise to you?

- Extremely important
- Very important
- Somewhat important
- Not so important
- Not at all important

* How many alcoholic drinks do you have each week?

- 0
- 1-4
- 5-8
- 9-12
- 13-16
- More than 16

* How often do you eat breakfast?

- Every day
- Three or more times a week
- Fewer than three times a week
- Only on weekends
- I rarely eat breakfast

* How often do you eat lunch?

- Every day
- Three or more times a week
- Fewer than three times a week
- Only on weekends
- I rarely eat lunch

* How often do you eat dinner?

- Every day
- Three or more times a week
- Fewer than three times a week
- Only on weekends
- I rarely eat dinner

* How often do you eat fast food?

- Every day
- A few times a week
- A few times a month
- Less than a few times a month
- Never

* In a typical day, how many microwavable or ready-made meals do you eat?

* How often do drink sugar-carbonated beverages?

- Every day (Several Times)
- Every day (Once)
- A few times a week
- A few times a month
- Less than a few times a month
- Never

* How often do eat sweet things e.g. sweets, chocolate, crisps?

- Every day (Several Times)
- Every Day (Once)
- A few times a week
- A few times a month
- Less than a few times a month
- Never

* How many **portions of fruit** do you eat each day? If you don’t know for certain, please provide an estimate.

- None
- 1 portion
- 2-3 portions
- 4-5 portions
- ≥ 6 portions

* How many **portions of vegetables** do you eat each day? If you don’t know for certain, please provide an estimate.

- None
- 1 portion
- 2-3 portions
- 4-5 portions
- ≥ 6 portions

* When is the last time that you saw a doctor?

- Less than 6 months ago
- 6 months ago to less than 1 year ago
- 1 year ago to less than 2 years ago
- 2 years ago to less than 3 years ago
- 3 or more years ago

* When is the last time that you saw a dentist?

- Less than 6 months ago
- 6 months ago to less than 1 year ago
- 1 year ago to less than 2 years ago
- 2 years ago to less than 3 years ago
- 3 or more years ago

* In general, how would you rate your overall mental or emotional health?

- Excellent
- Very good
- Good
- Fair
- Poor

* During the past 4 weeks, how bothered did you feel by emotional problems such as feeling anxious, depressed, irritable, or sad?

- Extremely bothered
- Very bothered
- Somewhat bothered
- Not so bothered
- Not at all bothered

* During the past 4 weeks, how disruptive were your physical health or emotional problems to your **normal social activities with family, friends, neighbours, or groups**?

- Extremely disruptive
- Very disruptive
- Somewhat disruptive
- Not so disruptive
- Not at all disruptive

* During the past 4 weeks, how disruptive were your physical health or emotional problems to your **normal professional activities**?

- Extremely disruptive
- Very disruptive
- Somewhat disruptive
- Not so disruptive
- Not at all disruptive

* During the past 4 weeks, how supported did you feel when you wanted or needed help at work or at home ? For example, if you felt lonely and wanted to talk to someone or you got sick.

- Extremely supported
- Very supported
- Somewhat supported
- Not so supported
- Not at all supported

* In my experience, surgery staff are treated fairly when they make mistakes.

- Strongly Disagree
- Disagree
- Neither Agree nor Disagree
- Agree
- Strongly Agree

* We have enough staff to handle the workload.

- Strongly Disagree
- Disagree
- Neither
- Agree
- Strongly Agree

* If asked, I would honestly tell patients how much sleep I had before their surgery.

- Strongly Disagree
- Disagree
- Neither Agree nor Disagree
- Agree
- Strongly Agree

* Error disclosure is promoted and implemented effectively in our profession.

- Strongly Disagree
- Disagree
- Neither Agree nor Disagree
- Agree
- Strongly Agree

* I have made **minor** work-errors as a result of fatigue.

- Strongly Disagree
- Disagree
- Neither Agree nor Disagree
- Agree
- Strongly Agree

* I have made **major** work-errors as a result of fatigue.

- Strongly Disagree
- Disagree
- Neither Agree nor Disagree
- Agree
- Strongly Agree

* How much exposure to COVID-19 positive patients have you had to this point?

- Significant exposure
- Relatively significant exposure
- Relatively insignificant exposure
- No exposure

* Have you personally contracted the COVID-19 virus?

- Yes
- No

In what ways has the COVID-19 pandemic impacted on your personal and/or professional performance?

Have you any other thoughts, comments or concerns?
